# Nature-based solutions to climate change

**DOI:** 10.1038/s41598-025-05678-7

**Published:** 2025-07-01

**Authors:** Denilson da Silva Bezerra

**Affiliations:** Federal University of Maranhão (UFMA), São Luís, Maranhão Brazil

**Keywords:** Climate-change impacts, Environmental impact

The twenty-first century is being marked by an intensification of the impacts of climate change^1^ and the growing loss of biodiversity^2^, both processes, unprecedented in the history of humanity and going far beyond scientific predictions. In this context, Nature-Based Solutions (NbS) emerge as a strategic, effective and sustainable approach to mitigate environmental impacts^3^, going from local to global interest, in addition to promoting the resilience of ecosystems/biomes and human societies.

NbS consist of the intelligent and sustainable use of natural processes (understanding the functioning of ecosystems/biomes, as well as the supply of their goods and services) to face socio-environmental challenges, such as carbon sequestration, firefighting, flood control, food and water security, among others^4^. This approach involves issues of ecosystem restoration and conservation, sustainable management of biodiversity, and even the recovery of the knowledge of traditional communities (fishing communities, indigenous populations, among others) about the functioning of natural systems, offering multiple interconnected benefits.

In this context, an important contribution of NbS in the fight against climate change lies in its ability to promote simultaneous mitigation and adaptation^5^. For example, forests, mangroves, native pastures, and wetlands can act as important carbon sinks, capturing carbon and other greenhouse gases from the atmosphere and storing them naturally in biomass and soil. At the same time, these ecosystems can act as natural barriers against extreme weather events, such as sea-level rise, floods, droughts, and landslides, functioning as true green infrastructures. Thus, investing in the restoration and conservation of these environments is a cost-effective strategy, promoting very efficient climate and social resilience; in addition, NbS can subsidize profitable economic activities based on sound ecological assumptions, such as in the case of “conventional” (for forests) and “blue” (for mangroves and other coastal ecosystems^6,7^) carbon credit activities.

The NbS are a powerful instrument to support the formulation of public policies, since they can promote concrete paths to align sustainable development and climate action^8,9^ with socio-environmental equity. Through policies that encourage payment for environmental services, regenerative agriculture, integrated water management and large-scale ecological restoration; It is important to mention that these actions can (and should) be incorporated into regulatory frameworks and territorial planning instruments, in a local, regional and global context.

The inclusion of NbS in national and international policies, such as the Nationally Determined Contributions (NDCs) established in the Paris Agreement (2017), is key to achieving ambitious and sustainable climate goals^10,11^. In 2025, humanity will once again have the opportunity to put NbS at the center of the climate discussion at the 30th Conference of the Parties (COP30) to the United Nations Framework Convention on Climate Change (UNFCCC) that will take place in November in the city of Belém (Brazil). COP30 is being called "the COP of nature", since, among the planned agenda points, one of the most important is the expectation that there will be the integration of the biodiversity agenda and the climate agenda, that is, to protect biodiversity as a way to increase climate resilience. And in this context, this collection brings important contributions to promote this ambitious agenda to be debated at COP30.

The articles published in this collection demonstrate the potential that NbS have for the integration of ecological, social and technological knowledge. Scientific research aimed at understanding the functioning of ecosystems and their services is essential for the effective implementation of these solutions. Climate modeling, biodiversity inventories, studies on ecological interactions, and ecosystem service assessments are fundamental tools that can help decision-makers around the world and at various levels (governments, civil society, and companies). In addition, the involvement of local communities and traditional knowledge strengthens the legitimacy and effectiveness of NbS, contributing to a more participatory and contextualized science; the following are some examples of research made available in our collection that readers from all over the world can have access to in an open way.

Carlson et al.^12^ warn that coral reefs provide important economic benefits to coastal companies around the world, supporting activities such as recreation and tourism, as well as providing protection to properties against storms. However, these benefits are at risk worldwide, as corals decline rapidly as a result of processes such as ocean acidification and rising water column temperatures, and there is a shortage of investment in restoration. Despite all the scientific knowledge about the ecosystem services provided by corals, it is not clear whether companies operating in the coastal zone perceive coral reef services as valuable or if they consider that they are also responsible for these very important ecosystems. Thus, the research presents an interesting methodological approach to estimate business perceptions about coral health and value and to identify characteristics correlated with business decisions to participate in coral restoration at differential pay levels.

Another interesting contribution is from Sunkur et al.^13^ who point out in their research, some of the main remote sensing techniques to promote monitoring of mangroves with the goal of their resilience to climate change. This contribution is important because mangroves are among the richest ecosystems in the world, providing valuable goods and services to millions of people while increasing the resilience of coastal communities against the impacts of climate change, especially island nations. The authors also warn that despite the available knowledge about the importance of mangroves, these ecosystems are severely affected by various anthropogenic activities, which increase their vulnerability to climate change.

Based on the premises presented in the paragraphs above, the editors and authors of this collection invite the scientific community in general, decision-makers, organized civil society, representatives of the private sector, students and others interested in the subject to honor the published research; not with the intention of "exhausting the debate", but rather, to further encourage discussions and the generation of new contributions on this very important theme for the twenty-first century.
